# FOXO4 and FOXD3 are predictive of prognosis in gastric carcinoma patients

**DOI:** 10.18632/oncotarget.8339

**Published:** 2016-03-24

**Authors:** Jing Li, Zhonghua Jiang, Fang Han, Shenxiang Liu, Xin Yuan, Jiandong Tong

**Affiliations:** ^1^ Department of Oncology, YangZhou No.1 People's Hospital, The Second Clinical School of Yangzhou University, Yangzhou, Jiangsu Province, China; ^2^ Department of Gastroenterology, The No.1 People's Hospital of Yancheng, Yancheng, China

**Keywords:** gastric cancer, FOXD3, FOXO4, survival analysis

## Abstract

Forkhead box (FOX) transcription factor family plays an important role in cancer growth and metastasis. This study aimed to determine the predictive ability of FOX genes in gastric carcinoma. A total of 360 patients with gastric from The Cancer Genome Atlas (TCGA) cohorts were collected in this study. The expression profile of FOX family were obtained from the TCGA RNAseq database. Clinicopathological characteristics, including age, gender, tumor node metastasis (TNM), tumor grade, and overall survival were collected. Univariate and multivariate Cox proportional hazards model were used to assess the risk factors for survival, and the results were further validated in in-house cohort. In the TCGA cohort, FOXO4 (HR = 0.613, 95%CI 0.452–0.832) and FOXD3 (HR = 1.704, 95%CI 1.212–2.397) were shown independently predictive of overall survival in gastric cancer after Cox proportional hazards analysis. The finding was validated in our in-house cohort, which demonstrated that both FOXO4 and FOXD3 were independent predictors for overall survival (FOXO4 high, HR: 0.445, 95%CI 0.277–0.715, *P* = 0.001, FOXD3 high, HR: 1.927, 95%CI 1.212–3.063, *P* = 0.006) and disease free survival (FOXO4 high, HR: 0.628, 95%CI 0.420–0.935, *P* = 0.022, FOXD3 high, HR: 1.698, 95%CI 1.136–2.540, *P* = 0.010). Collectively, FOX family paly critical roles in gastric cancer, and FOXO4 and FOXD3 were identified as independent prognostic factors for survival outcomes of gastric cancer. Further functional study is needed to understand more about FOX family in gastric cancer.

## INTRODUCTION

Gastric cancer represents a major cause of cancer mortality because of its poor prognosis [1]. The only potentially curative treatment for gastric cancer is complete resection (R0). However, despite aggressive surgical intervention, more than 50% of patients undergoing radical resection will experience disease recurrence, usually in the form of metastatic disease. The development of metastatic disease is almost invariably lethal, and it is estimated in 2015 that over 10, 720 individuals in the United States will perish from metastatic gastric cancer in the United States [2]. Thus, a better understanding of the underlying mechanisms that promote the pathogenesis and progression of gastric cancer is urgently needed.

Forkhead box (FOX) transcription factors are a large evolutionarily conserved family of transcriptional regulators that share a highly conserved winged helix DNA binding domain. Outside of this domain, FOX family have diverged into sub-families and incorporated a variety of other domains conferring on them a plethora of functions [3]. FOX family has been recently reported to be involved in various cancer progression and metastasis [4–9], and they have been found operating as both oncogenes and tumor suppressors via a variety of mechanisms. For example, FoxM1c induces EMT by activating the uPA system/Slug pathway [5]. FoxC2 promote epithelial mesenchymal transition (EMT) and colorectal cancer metastasis through the Akt/GSK-3β/Snail Pathway [7]. Loss of FOXA1 is associated with high grade, late stage bladder cancer and increased tumor proliferation [8]. FOXO3a modulates WNT/β-catenin signaling and suppresses EMT in prostate cancer cells [9]. But the potential role of FOX family in gastric cancer and its biological functions on the initiation, progression, and outcome of the disease remains not fully understand.

To describe the characteristics of FOX genes in gastric cancer in depth, we analyzed all FOX family genes in 360 gastric cancer cases from The Cancer Genome Atlas (TCGA) cohort and an additional 226 cases in-house validated cohort. We also examined their associations with clinicopathologic characteristics and survival outcomes of gastric tumors.

## RESULTS

### Clinical factors in TCGA and validated cohorts

In the TCGA cohort, the median age of these 360 gastric patients was 65, ranging from 30 to 90 years old. Two hundred thirty-four (65.0%) were male patients and 126 (35.0%) were female patients. Gender, age of diagnosis, TNM, tumor grade, are shown in Table 1. The median length of follow-up was 16 months (range, 1 month-124 months) and 226 patients had died at the end of follow-up.

**Table 1:** Clinical characteristics of patients with gastric in TCGA and validated cohort

Variable		TCGA		Validated cohort	
		*N*	%	*N*	%
Sex					
	male	234	65.0	122	54.0
	female	126	35.0	104	46.0
Age		65	30–90	58	19–82
Primary site					
	Antrum/Distal	137	38.1	84	37.2
	Cardia/Proximal	48	13.3	77	34.1
	Fundus/Body	132	36.7	44	19.5
	Gastroesophageal Junction	37	10.3	21	9.3
	Unspecific	6	1.7	0	0
Grade					
	G1/G2	133	36.9	96	42.5
	G3	218	60.6	126	55.8
	Gx	9	2.5	4	1.8
T stage					
	T1	17	4.7	3	1.3
	T2	70	19.4	32	14.2
	T3	167	46.4	100	44.2
	T4	105	29.2	91	40.3
	Tx	1	0.3	0	0
N stage					
	N0	113	31.4	62	27.4
	N1	94	26.1	50	22.1
	N2	72	20.0	50	22.1
	N3	75	20.8	64	28.3
	Nx	6	1.7	0	0
M stage					
	M0	328	91.1	226	100
	M1	18	5.0	0	0
	Mx	14	3.9	0	0

In the validated cohort, the median age of these 226 gastric cancer patients was 58, ranging from 19 to 82 years old. One hundred twenty-two (54.0%) were male and 104 (46.0%) were female patients. The expression levels of FOX family genes (FOXD3, FOXO4) in this cohorts were nearly normal distributed (data not shown); therefore, we divided the two cohorts into low or high expression groups according to median expression level. At last follow up, ninety-nine patients were relapsed and 76 were died. The median follow-up time of this cohort was 32 months. The characteristics of the samples are shown in Table 1.

### FOXD3 and FOXO4 were independent prognostic factors for OS in the TCGA cohort

In univariate Cox proportion hazard ratio analysis, age, tumor(T) stage, Node(N) stage, metastasis(M) stage, FOXD3, FOXO4 and FOXS1 expression were significantly associated with prognosis in terms of OS of patients with gastric cancer in the TCGA cohorts (*p* < 0.05, Table 2). A reduced model was used in the multivariate Cox analysis, which means only variables that were significantly correlated with prognosis in univariate Cox proportion hazard ratio (HR) analysis were included in the next step. Multivariate analysis after adjustment for all the potential prognostic factors demonstrated that age (HR = 1.036, 95% CI 1.016–1.055, *P* < 0.001), T stage (HR = 1.326, 95% CI 1.028–1.709, *P* = 0.030), N stage (HR = 1.271, 95% CI 1.077–1.500, *P* = 0.005), FOXD3 (HR = 1.704, 95% CI 1.212–2.397, *P* = 0.002), and FOXO4 (HR = 0.613, 95% CI 0.452–0.832, *P* = 0.002) were independent predictors of OS (Table 2).

**Table 2:** Univariate and multivariate Cox proportional hazards analysis of FOX gene expression and overall survival for patients with gastric cancer in the TCGA cohort

	Univariate analysis		Multivariate analysis	
Factor	HR (95% CI)	*P*	HR (95% CI)	*P*
Gender	0.681 (0.457–1.016)	0.060		
Age	1.020 (0.585–1.792)	**0.014**	1.036 (1.016–1.055)	**< 0.001**
T category	1.375 (1.094–1.727)	**0.007**	1.326 (1.028–1.709)	**0.030**
N stage	1.300 (1.113–1.518)	**0.001**	1.271 (1.077–1.500)	**0.005**
M stage	1.590 (1.132–2.234)	**0.007**	1.384 (0.978–1.959)	0.066
Grade	1.313 (0.933–1.848)	0.119		
Tumor location	0.971 (0.828–1.139)	0.721		
FOXA1	0.985 (0.870–1.116)	0.815		
FOXA2	0.979 (0.864–1.111)	0.746		
FOXA3	0.884 (0.773–1.013)	0.078		
FOXC1	0.986 (0.845–1.152)	0.862		
FOXC2	1.139 (0.899–1.442)	0.282		
FOXD1	0.955 (0.824–1.106)	0.536		
FOXD2	0.818 (0.640–1.044)	0.107		
FOXD3	1.419 (1.007–1.999)	**0.045**	1.704 (1.212–2.397)	**0.002**
FOXD4	0.564 (0.284–1.117)	0.101		
FOXF1	1.066 (0.923–1.239)	0.370		
FOXF2	0.990 (0.848–1.157)	0.903		
FOXH1	1.067 (0.861–1.323)	0.552		
FOXI1	0.694 (0.413–1.164)	0.167		
FOXJ1	0.971 (0.866–1.089)	0.616		
FOXJ2	0.980 (0.668–1.438)	0.919		
FOXJ3	0.897 (0.613–1.313)	0.575		
FOXK1	1.001 (0.751–1.336)	0.989		
FOXK2	0.972 (0.657–1.437)	0.887		
FOXL1	1.138 (0.882–1.468)	0.320		
FOXL2	0.998 (0.639–1.559)	0.994		
FOXM1	1.000 (0.835–1.197)	0.998		
FOXN2	0.948 (0.665–1.350)	0.767		
FOXN3	1.179 (0.913–.522)	0.208		
FOXO1	1.008 (0.753–1.349)	0.958		
FOXO3	1.174 (0.891–1.547)	0.255		
FOXO4	0.681 (0.513–0.904)	**0.008**	0.613 (0.452–0.832)	**0.002**
FOXP1	1.018 (0.751–1.380)	0.910		
FOXP2	1.144 (0.961–1.362)	0.131		
FOXP3	0.807 (0.629–1.035)	0.092		
FOXP4	0.938 (0.747–1.177)	0.581		
FOXQ1	0.919 (0.817–1.035)	0.165		
FOXS1	1.219 (1.008–1.474)	**0.041**	1.180 (0.957–1.455)	0.121

Abbreviations: CI, confidence interval; HR, hazard ratio.

Bold type indicates statistical significance.

### FOXD3 and FOXO4 expressions were prognostic factors for OS and DFS in the validated cohort

These results should be treated with caution because they could be biased by confounding factors that were not specified in TCGA database, such as lymphovascular invasion, perineural invasion and quality of surgery (palliative resection or radical resection). To evaluate the reliability of TCGA results, we validated the results in 226 in-house eligible patients. Patient demographics and pathological features are summarized in Table 1. Likewise, we divided the cohort into low- and high expression groups according to median expression level.

**Figure 1:**
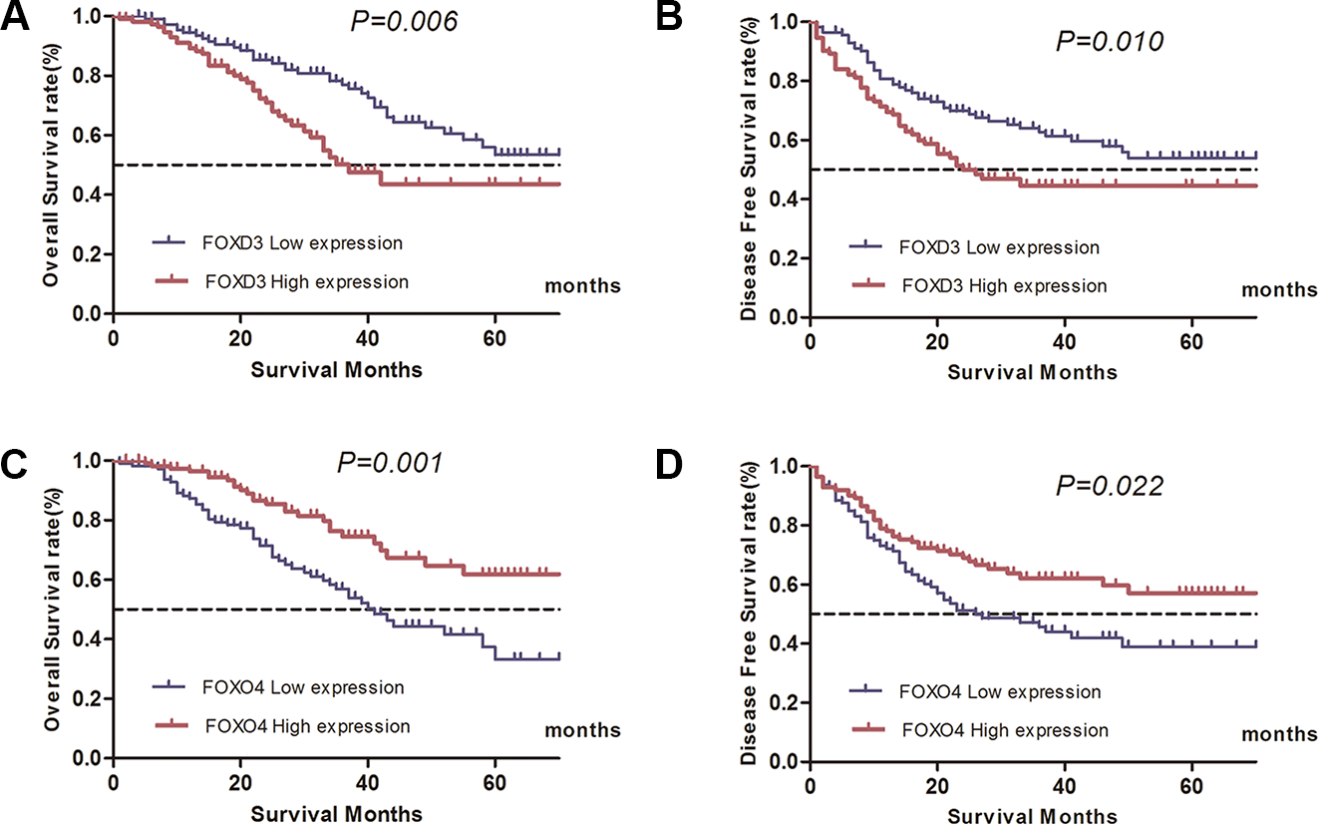
Influence of FOXO4 and FOXD3 expression patterns on overall survival and disease-free survival by Kaplan-Meier analyses in validated cohort (**A**) FOXD3, OS: χ^2^ = 7.920, *P* = 0.006; (**B**) FOXD3, DFS: χ^2^ = 6.869, *P* = 0.010; (**C**) FOXO4, OS: χ^2^ = 11.786, *P* = 0.001; (**D**) FOXO4, DFS: χ^2^ = 5.378, *P* = 0.022.

χ^2^ tests demonstrated that FOXO4 mRNA expression level was inversely correlated with T stage (*P* < 0.01), N stage (*P* = 0.027), while FOXD3 expression was positively correlated with T stage (*P* = 0.043) (Supplementary Table S1). Five year OS and DFS were 61.5%, 37.3% and 56.5%, 34.6% for FOXO4 high and low expression, low FOXO4 expression was associated with poor prognosis for both OS (log-rank test, *p* = 0.001) and DFS (log-rank test, *p* = 0.022), 5-year OS and DFS were 43.1%, 55.9% and 37.2% and 46.7% for low and high expression of FOXD3, high level of FOXD3 expression was correlated with poor prognosis for OS (log-rank test, *p* = 0.006) and DFS (log-rank test, *p* = 0.010) (Table 4). The Kaplan–Meier curves are shown in Figure 1.

**Table 3:** Univariate and multivariate Cox proportional hazards analysis of FOX gene expression and overall survival for patients with gastric cancer in the validated cohort

Factor	Univariate analysis		Multivariate analysis	
	HR (95% CI)	*P*	HR (95% CI)	*P*
Gender	0.750 (0.475–1.183)	0.216		
Age	1.238 (0.789–1.945)	0.353		
T category	1.735 (1.258–2.393)	**0.001**	1.215 (0.801–1.843)	0.359
N stage	1.559 (1.272–1.909)	**< 0.001**	1.373 (1.057–1.784)	**0.017**
Grade	1.737 (1.096–2.751)	**0.019**	1.705 (1.052–2.762)	**0.030**
Lymphovascular invasion	1.808 (1.137–2.875)	**0.012**	2.174 (1.348–3.506)	**0.001**
Perineural invasion	1.930 (1.181–3.154)	**0.009**	1.384 (0.837–2.290)	0.206
Tumor location	0.170 (0.962–1.462)	0.116		
FOXO4	0.445 (0.277–0.715)	**0.001**	0.281 (0.168–0.469)	**< 0.001**
FOXD3	1.927 (1.212–3.063)	**0.006**	2.576 (1.553–4.274)	**< 0.001**

Abbreviations: CI, confidence interval; HR, hazard ratio.

Bold type indicates statistical significance.

**Table 4:** Univariate and multivariate Cox proportional hazards analysis of FOX gene expression and disease free survival for patients with gastric cancer in the validated cohort

Factor	Univariate analysis		Multivariate analysis	
	HR (95% CI)	*P*	HR (95% CI)	*P*
Gender	0.832 (0.559–1.238)	0.365		
Age	1.268 (0.853–1.885)	0.240		
T category	1.827 (1.347–2.478)	**< 0.001**	1.374 (0.956–1.975)	0.086
N stage	1.454 (1.223–1.728)	**< 0.001**	1.306 (1.059–1.610)	**0.012**
Grade	1.829 (1.220–1.743)	**0.003**	1.608 (1.059–2.441)	**0.026**
Lymphovascular invasion	1.595 (1.049–2.424)	**0.029**	1.719 (1.121–2.638)	**0.013**
Perineural invasion	2.137 (1.390–3.285)	**0.001**	1.602 (1.029–2.493)	**0.037**
Tumor location	1.116 (0.938–1.328)	0.216		
FOXO4	0.628 (0.420–0.935)	**0.022**	0.451 (0.295–0. 691)	**< 0.001**
FOXD3	1.698 (1.136–2.540)	**0.010**	1.966 (1.282–3.014)	**0.002**

Abbreviations: CI, confidence interval; HR, hazard ratio.

Bold type indicates statistical significance.

Besides, in univariate Cox proportion hazard ratio analysis, tumor stage, N stage, tumor grade, present of lymphovascular invasion and perineural invasion were all significantly associated with poor prognosis in terms of OS and DFS (*P* < 0.05, Tables 3, 4). Multivariate analysis after adjustment for all the potential prognostic factors indicated that FOXO4 and FOXD3 expression level were the two strong predictors of OS (FOXO4 high, HR: 0.281, 95% CI 0.168–0.469, *P < 0.001*, FOXD3 high, HR: 2.576, 95% CI 1.553–4.274, *P* < 0.001) and DFS (FOXO4 high, HR: 0.451, 95% CI 0.295–0.691, *P* < 0.001, FOXD3 high, HR: 1.966, 95% CI 1.282–3.014, *P* = 0.002) (Tables 3, 4).

## DISCUSSION

In this study, to our knowledge, for the first time, we comprehensive demonstrated that FOX family correlated with OS and DFS of gastric cancer patients. Members of this family, especially FOXO4 and FOXD3, are two independent prognostic factors for OS and DFS of gastric cancer patients.

FOX proteins are a family of transcription factors that play important roles in regulating the expression of genes involved in cell growth, proliferation, differentiation, and longevity. Many FOX proteins are important to embryonic development [10, 11]. FOX proteins have pioneering transcription activity by being able to bind condensed chromatin during cell differentiation processes [12]. Despite the highly conserved FOX DNA-binding domain, Fox protein regulation and function vary significantly between families [3]. For examples, ectopic expression of the FOXC2 accelerates the development, proliferation and growth of tumors in colorectal cancer [7]. By contrast, activation of the FoxO family of proteins is associated with cell cycle arrest and the induction of apoptosis [13, 14]. Given that FOX family genes control these essential developmental and homeostatic processes, it is not surprise that a loss or gain of Fox function can alter cell fate and lead to tumorigenesis. Despite the fact that our knowledge of FOX transcription factors is still in its infancy, several FOX subfamilies such as FOXA, FOXC, FOXM, FOXP, and FOXO have been linked to tumorigenesis and the progression of some cancers [3]. Here, we investigated the relevant of FOX genes and gastric cancer comprehensively.

The FOXO transcription factor family contains three members in mammalian cells, including FOXO1, FOXO3, and FOXO4. FOXO family members play important roles in cell cycle progression [14, 15], apoptosis [13], oxidative stress [14], DNA repair [16] and drug sensitivity [17]. Importantly. Loss of FOXO function has been observed in prostate cancer [18], non-small lung cancer [19], nasopharyngeal carcinoma [20], and breast cancer [21]. Recently study indicated that FOXO4 is down-regulated and inhibits tumor proliferation and metastasis in gastric cancer [22]. In light of these important studies, it is likely that FOXO4 has a tumor suppressive function that is inactivated in tumorigenesis. Our study give a new insight of FOXO4 gene functional role in gastric cancer and found it was an important prognostic biomarker in gastric.

FOXD3, one member of the FOXD transcription factor family, is originally identified in embryonic stem cells [23] and plays crucial roles in the neural crest development and stem cell biology through specifying the cell lineage [24, 25]. FOXD3 knockout results in early embryonic death in mice [24]. These indicate that FOXD3 plays a primary role in embryonic development, and it is interesting to investigate the potential roles of FOXD3 in the tumors. To date, the results seems controversies, previous study indicate that FOXD3 exhibits tumor suppressive activity that affects the growth, aggressiveness and angiogenesis of neuroblastoma [26]. Promoter hypermethylation could slicing FOXD3 expression and significantly promotor gastric cancer cell proliferation and invasion [27]. However, in mutant B-RAF melanoma cells, adaptive upregulation of FOXD3 can cause resistance to PLX4032/4720 (a target therapy regent)-induced cell death. In our study, the FOXD3 expression level was determined at transcriptional level, as the RNAseq in TCGA database and qRT-PCR analysis in validated database, we confirmed low FOXD3 expression was favorable prognostic factor in gastric. Our results seems controversies with previous functional study *in vitro* [27], and it will deserved further study.

A major strength of this study is that the information was obtained from two independent populations with a relative long-time follow up, but there are certain limitations. The prognosis of gastric is affected by many factors such as patients' immune status, surgical techniques, and response to adjuvant therapy, so biomarkers from a single gene family is not enough. Second, the data from TCGA was public available and patients' number was large, which make our results reliable, however, for the complicated interactive network and signaling pathways *in vivo*, the FOX family genes which were not validated as biomarkers in this study may also play critical role in gastric cancer, and it need further study. In addition, information regarding disease recurrence and metastasis is unavailable in the TCGA cohort, and therefore only OS could be evaluated.

In conclusion, FOX family paly critical roles in gastric cancer, and FOXO4 and FOXD3 were identified as independent prognostic factors for survival outcomes of gastric cancer. Further functional study is needed to understand more about FOX family in gastric cancer.

## MATERIALS AND METHODS

### Patients and samples

This study received Institutional Review Board approval from Yangzhou No.1 people's hospital. Written informed consent was obtained from all subjects. The methods were carried out in accordance with the approved guidelines.

For the TCGA cohort, FOX genes expression and clinical data of TCGA database are available from the website of Cancer Genomics Browser of University of California Santa Cruz (UCSC) (https://genome-cancer.ucsc.edu/). Eleven members of the FOX family were excluded from the study for extremely low mRNA copy number (FOXB1, FOXE1, FOXE3, FOXI2, FOXN1, FOXN4), or the copy number was 0 in more than 2/3 patients (FOXB2, FOXG1, FOXI3, FOXR1, FOXR2). As results, thirty-two members of the FOX family are included in the database as is shown in Table 1. Other inclusion criteria were: patients with no pretreatment, with fully characterized tumors and intact overall survival (OS) information. Follow-up was completed on Dec 21, 2014.

The validated cohort consists of 226 patients with histologically confirmed invasive gastric cancer who had undergone radical surgical resection between January 1, 2003 and December 31, 2009. All patients received no pretreatment, and only patients without any evidence of metastasis at the time of diagnosis were enrolled. Demographic and clinical characteristics, such as age, sex, age at initial diagnosis, and stage at diagnosis (tumor, node, metastasis [TNM] classification) were obtained from electronic records and summarized in Table 1.

### RNA extraction, reverse transcription, and qRT-PCR analysis

Total RNA was isolated from 226 gastric cancer samples using TRIzol^®^ reagent (15596026, Invitrogen). A PrimeScript™ RT Master Mix (Perfect Real Time) kit (RR036A, Takara) was used to synthesize first-strand cDNA from total RNA. After that, SYBR Green real-time PCR assays were performed using an ABI 7900HT (Applied Biosystems, USA). The expression level of RNA was normalized to the level of GAPDH. The primers for RT-PCR analysis were synthesized by Huagene (Shanghai, People's Republic of China), the sequences of which are shown in Supplementary Table S2.

### Statistical analysis

All statistical analysis was performed using SPSS software (version 21.0, IBM Corp., Armonk, NY, USA). Independent *t*-tests (for continuous variables) and Pearson's χ2 tests (for categorical variables) were used. The cut-point of FOX genes mRNA expression was defined as the median. The overall survival (OS) was defined as the time from surgery to death due to any cause. The disease-free survival (DFS) was defined as the time of surgery to tumor recurrence, progression or metastasis in localized gastric cancer. The difference in survival between the groups was compared by the log rank test. Variables that seemed to be significantly associated with survival on univariate analysis were entered into multivariate analysis, which was performed with Cox proportional hazard model. Patients without events or death were recorded as censored at the time of last follow-up. A two-sided *P*-value < 0.05 was considered to indicate statistical significance.

## SUPPLEMENTARY MATERIALS TABLES


