# Supplementary material for: FOXO4 and FOXD3 are predictive of prognosis in gastric carcinoma patients
Source: Oncotarget. 2016 Mar 24;7(18):25585–92. doi: 10.18632/oncotarget.8339 (PMC5041928; doi:10.18632/oncotarget.8339)
Supplement: Supplementary file 1 [file oncotarget-07-25585-s001.pdf]

FOXO4 and FOXD3 are predictive of prognosis in gastric carcinoma patients

Supplementary Materials

Supplementary Table S1: Comparison of baseline clinical characteristics based on FOXO4 and FOXD3 mRNA expression levels

Variable		FOXO4			FOXD3		
		Low (%)	High (%)	*P*	Low	High	*P*
Sex				0.183			0.790
	male	53 (46.9)	63 (55.8)		57 (50.4)	59 (52.2)	
	female	60 (53.1)	50 (44.2)		56 (49.6)	54 (47.8)	
Age				0.903			0.428
	≥ 60	59 (52.2)	63 (55.8)		56 (49.6)	66 (58.4)	
	< 60	54 (48.8)	50 (44.2)		57 (50.4)	47 (41.6)	
Primary site				0.925			0.706
	Antrum/Distal	43 (38.1)	41 (36.3)		42 (37.2)	42 (37.2)	
	Cardia/Proximal	29 (34.5)	38 (33.6)		40 (35.4)	37 (32.7)	
	Fundus/Body	20 (17.7)	24 (21.2)		23 (20.4)	21 (18.6)	
	Gastroesophageal Junction	11 (9.7)	10 (8.8)		8 (7.1)	13 (11.5)	
Grade				0.095*			0.549*
	G1/G2	40 (35.4)	56 (49.6)		50 (44.2)	46 (40.7)	
	G3	71 (62.8)	55 (48.7)		62 (54.9)	64 (56.6)	
	Gx	2 (1.8)	2 (1.8)		1 (0.9)	3 (2.7)	
T stage							0.043
	T1/T2	6 (5.3)	29 (25.7)	< 0.001	23 (20.4)	12 (10.6)	
	T3/T4	107 (94.7)	84 (74.3)		90 (79.6)	101 (89.4)	
N stage				0.027			0.152
	N0	24 (21.2)	38 (33.6)		34 (30.1)	28 (24.8)	
	N1	27 (23.9)	23 (20.4)		28 (24.8)	22 (19.5)	
	N2	33 (29.2)	17 (15.0)		18 (15.9)	32 (28.3)	
	N3	29 (25.7)	35 (31.0)		33 (29.2)	31 (27.4)	
Lymphovascular invasion				0.881			0. 653
	Negative	83 (73.5)	82 (72.6)		81 (71.7)	84 (74.3)	
	Positive	30 (26.5)	31 (27.4)		32 (28.3)	29 (25.7)	
Perineural invasion				0.413			0.251
	Negative	87 (77.0)	92 (81.4)		93 (82.3)	86 (76.1)	
	Positive	26 (23.0)	21 (18.6)		20 (17.7)	27 (23.9)	

*Fisher's exact test.

Supplementary Table S2: The primers for RT-PCR analysis

primers	Sequences (5′to3′)
FOXO4F	CCGGCAAAAGCTCTTGGTG
FOXO4R	GGTCCACATATCGGCTTCTTCA
FOXD3F	ATTCTTAGCGTGCCACTCTCCGG
FOXD3R	CTGGAGGTGCATTTGTTGCTCGA
FOXS1F	AGTGGCATCTACCGCTACATC
FOXS1R	CACCTTGACAAAGCACTCGT
actinF	CTACGTCGCCCTGGACTTCGAGC
actinR	GATGGAGCCGCCGATCCACACGG
